# Omega-3 Fatty Acids and Vitamin D Decrease Plasma T-Tau, GFAP, and UCH-L1 in Experimental Traumatic Brain Injury

**DOI:** 10.3389/fnut.2021.685220

**Published:** 2021-06-04

**Authors:** Angus G. Scrimgeour, Michelle L. Condlin, Andrei Loban, James C. DeMar

**Affiliations:** ^1^Military Nutrition Division, US Army Research Institute of Environmental Medicine, Natick, MA, United States; ^2^Blast-Induced Neurotrauma Branch, Center for Military Psychiatry and Neuroscience Research, Walter Reed Army Institute of Research (WRAIR), Silver Spring, MD, United States

**Keywords:** mild traumatic brain injury, exercise, omega-3 fatty acids, vitamin D_3_, neurotrauma biomarkers

## Abstract

Traumatic brain injury (TBI) results in neuronal, axonal and glial damage. Interventions targeting neuroinflammation to enhance recovery from TBI are needed. Exercise is known to improve cognitive function in TBI patients. Omega-3 fatty acids and vitamin D reportedly reduce inflammation, and in combination, might improve TBI outcomes. This study examined how an anti-inflammatory diet affected plasma TBI biomarkers, voluntary exercise and behaviors following exposure to mild TBI (mTBI). Adult, male rats were individually housed in cages fitted with running wheels and daily running distance was recorded throughout the study. A modified weight drop method induced mTBI, and during 30 days post-injury, rats were fed diets supplemented with omega-3 fatty acids and vitamin D_3_ (AIDM diet), or non-supplemented AIN-76A diets (CON diet). Behavioral tests were periodically conducted to assess functional deficits. Plasma levels of Total tau (T-tau), glial fibrillary acidic protein (GFAP), ubiquitin c-terminal hydrolase L1 (UCH-L1) and neurofilament light chain (NF-L) were measured at 48 h, 14 days, and 30 days post-injury. Fatty acid composition of food, plasma, and brain tissues was determined. In rats exposed to mTBI, NF-L levels were significantly elevated at 48 h post-injury (*P* < 0.005), and decreased to levels seen in uninjured rats by 14 days post-injury. T-tau, GFAP, and UCH-L1 plasma levels did not change at 48 h or 14 days post-injury. However, at 30 days post-injury, T-tau, GFAP and UCH-L1 all significantly increased in rats exposed to mTBI and fed CON diets (*P* < 0.005), but not in rats fed AIDM diets. Behavioral tests conducted post-injury showed that exercise counteracted cognitive deficits associated with mTBI. The AIDM diets significantly increased docosahexaenoic acid levels in plasma and brain tissue (*P* < 0.05), and in serum levels of vitamin D (*P* < 0.05). The temporal response of the four injury biomarkers examined is consistent with studies by others demonstrating acute and chronic neural tissue damage following exposure to TBI. The anti-inflammatory diet significantly altered the temporal profiles of plasma T-tau, GFAP, and UCH-L1 following mTBI. Voluntary exercise protected against mTBI-induced cognitive deficits, but had no impact on plasma levels of neurotrauma biomarkers. Thus, the prophylactic effect of exercise, when combined with an anti-inflammatory diet, may facilitate recovery in patients with mTBI.

## Introduction

Traumatic brain injury (TBI) constitutes a critical health problem. More than 69 million new cases occur worldwide each year (1), accounting for upwards of a million deaths (roughly 2,700 deaths per day) and a global financial burden of US $400 billion (2, 3). TBI, either isolated or compounded with other injuries (i.e., polytrauma), is also a major cause of short- and long-term disability. Despite decades of rigorous preclinical studies and hundreds of randomized controlled clinical trials testing neuroprotective drug approaches with different pathophysiological targets (4, 5), effective evidence-based therapeutics for TBI-induced neuropathologies, behavioral, psychiatric, emotional, and other cognitive impairments are lacking (2, 6, 7). This has made exploration of alternative TBI therapies a priority of the health care system.

Cognitive and neurological impairments are prevalent consequences of mild TBI (mTBI), which constitutes more than 60% of brain injuries in civilian populations (8). These cognitive deficits are frequently related to hippocampal dysfunction (9), and have been reliably reproduced in animals models of TBI (10–12). Significant adverse effects on balance and cognition develop within the first 24 h following mTBI (13), and often extend well into the first week following injury (14, 15). These deficits improve and typically resolve within 3 months of injury (16); however, ~20% of cases of mTBI report symptoms 1 year after injury (17). A differential diagnosis in the initial days following mTBI is a challenging clinical situation, largely due to the absence of reliable prognostic or early detection biomarkers. The cause of persistent symptoms is likely multifactorial (18). Efforts to identify more sensitive, reliable, and/or objective indicators of brain injury and recovery have focused on biomarkers, microstructural changes in white matter, neurometabolic alterations and differences in neurochemistry, and functional and physiological changes in neural networks. Recently, several specific protein biomarkers were identified in the context of mTBI, including total tau (T-tau), glial fibrillary acidic protein (GFAP), ubiquitin carboxy-terminal hydrolase-L1 (UCH-L1), and neurofilament light chain (NF-L) (19–21). Previous studies and consensus statements (22, 23) have reported high sensitivity and decisive predictive value of these as biomarkers of injury severity, in regard to cellular origin and temporal trajectories, which will help improve outcome prediction.

As mentioned above, despite given the prevalence of TBI-related disabilities, effective treatment strategies are lacking and a recent review of therapies for neurobehavioral and cognitive recovery after experimental TBI suggesting that a multi-targeted combination of therapies could be more beneficial than monotherapies because multiple and incompletely understood cellular mechanisms of action are at play. The report indicates that 46% of studies exhibited an additive or synergistic (positive) effect, vs. only 19% reported a negative interaction (6). Nutrition interventions for both prevention and treatment of TBI symptomatologies have been reported (24–26). While several therapies, including vitamins (B_2_, B_3_, B_6_, B_9_, C, D, and E) and nutrients (arginine, carnitine, magnesium, omega-3 fatty acids, resveratrol, selenium, and zinc) have demonstrated efficacy in preclinical studies, using rats and mice, but investigations of polytherapy treatments have not been conducted.

In preclinical studies, omega-3 fatty acid enriched dietary supplements (e.g., fish oil) provided protection against reduced plasticity and impaired learning after TBI (27–29). For example, in rats supplemented with docosahexaenoic acid (DHA, 12.4%) and eicosapentaenoic acid (EPA, 13.5%) for 4 weeks prior to mild lateral fluid percussion induced TBI, neuronal function and plasticity were maintained post injury, and markers of oxidative stress were significantly reduced (27). Vitamin D supplementation has also demonstrated neuroprotective properties in multiple preclinical models of TBI. In studies on rats, vitamin D monotherapy (5 μg/kg, injected intraperitoneally 1 h post-injury) reduced inflammation and neuronal injury following TBI (30, 31), and vitamin D deficiency was shown to exacerbate the post-TBI inflammatory response (32). Based on evidence that demonstrated individual interventions with omega-3 fatty acids and vitamin D in restoring/repairing neurotrauma post-TBI, we hypothesized that an anti-inflammatory diet (AIDM) incorporating multiple corresponding nutrients and implemented post-injury would enhance recovery of cognitive functions, and reduce/minimize plasma levels of T-tau, GFAP, UCH-L1, and NF-L in a rat model of TBI. Thus, the aim of the present study was to evaluate the effect of an AIDM diet on these four neurotrauma biomarkers. Both behavioral deficits (motor, memory and anxiety assessments) and neurotrauma biomarkers were evaluated in the context of the nutritional intervention to help address resiliency and recovery.

## Methods

### Experimental Design

**Figure 1:**
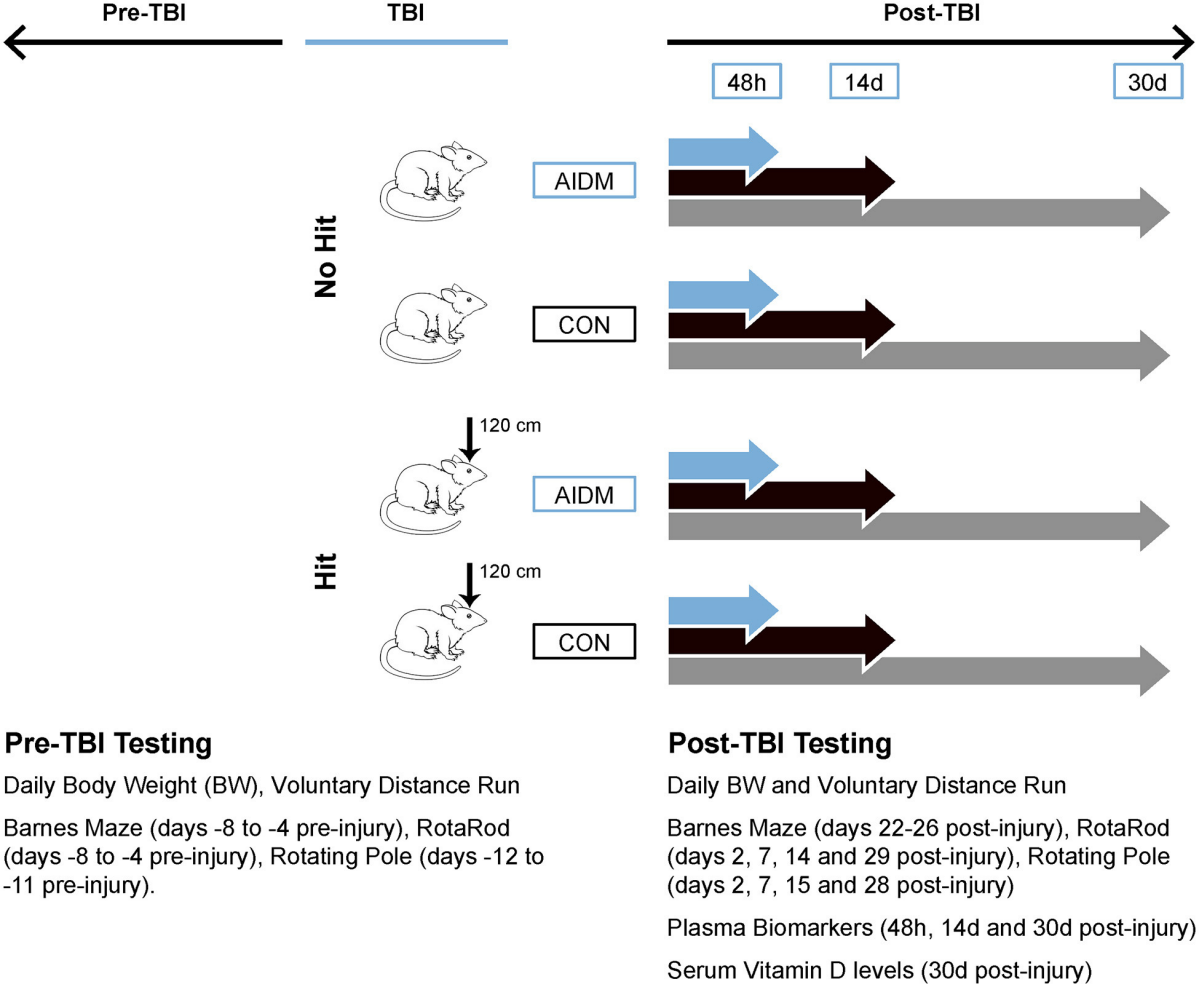
Workflow diagram of study's experimental design. Using a 2 x 2 design, male, Wistar-Han rats were randomly assigned to 1 of 4 groups: TBI-exposed (Hit) or non-TBI (No Hit), and either anti-inflammatory (AIDM) or non-supplemented AIN-76A (CON) diets following exposure to TBI. At select time points (48 h, 14 days, and 30 days) post-injury, plasma and brain (frontal cortex) tissues were harvested. Physiological and behavioral measures pre- and post-TBI are listed in the inset. 120 cm, height from which 500 g slug was dropped; BW, body weight; TBI, traumatic brain injury.

A total of 120 rats were randomly assigned to the following four groups: TBI-exposed (Hit) or non-TBI (No Hit), and either control (CON) or anti-inflammatory (AIDM) diets during their recovery period. The experimental data from the CON and AIDM naïve groups was collected during a pilot study conducted before those involving the Hit or No Hit groups. No behavioral assessments were made during the pilot study, the purpose of which was to ensure the nutrient composition of the experimental AIDM was acceptable to the rats, and that it produced the desired increase in tissue nutrient concentrations (assayed using same methods described below for Hit and No Hit rats) in rats that consumed it, compared to rats who consumed the standard diet (see Table 1). A battery of behavioral assessments were performed pre- and periodically post-injury, as described below (and depicted in Figure 1), including distance run on the voluntary running wheels, the Rotarod test, a rotating pole test, and the Barnes Maze (BM) test. For behavioral measures, 12 rats were included in each group. For the analysis of neurotrauma biomarkers, six rats in each group were sacrificed at 48 h post-injury (acute), while 12 rats in each group were sacrificed at the chronic time points (14 and 30 days), post-injury. All efforts were made to minimize animal pain and distress and the number of animals used. This study was approved by the Institutional Animal Care and Use Committee at USARIEM. In conducting the research described in this report, the investigators adhered to the Guide for Care and Use of Laboratory Animals (34).

**Table 1:** Pilot study data of fatty acid composition of rat diets, and resultant plasma and brain values in non-injured animals after 30 days on CON or AIDM diets.

**Ingredient**	**CON drink consumed/day^**α**^**	**AIDM drink consumed/day^**α**^**	**CON chow (mg/day)^**#**^**	**AIDM chow (mg/day)^**#**^**	**CON rat plasma (% of total)**	**AIDM rat plasma (% of total)**	**CON rat liver (% of total)**	**AIDM rat liver (% of total)**	**CON rat brain (% of total)**	**AIDM rat brain (% of total)**
18:2 (n-6) linoleic acid	<0.24 mg	13.64 mg	629.1	551.5	30.41 ± 2.33	29.35 ± 2.92	21.20 ± 3.12	23.93 ± 2.65	0.95 ± 0.28	0.82 ± 0.07
18:3 (n-3) α-linolenic acid	<0.24 mg	0.62 mg	<0.007	<0.007	1.56 ± 0.38	1.54 ± 0.35	0.50 ± 0.31	0.46 ± 0.14	ND	ND
20:4 (n-6) arachidonic acid	<0.24 mg	1.74 mg	<0.007	<0.007	0.57 ± 0.51	0.68 ± 0.37	1.16 ± 1.45	0.48 ± 0.83	1.14 ± 1.61	0.49 ± 0.87
20:5 (n-3) eicosapentaenoic acid	<0.24 mg	45.94 mg	<0.007	<0.007	ND	1.25 ± 1.26*	0.20 ± 0.14	1.39 ± 0.52*	ND	ND
22:5 (n-3) docosapentaenoic acid	<0.24 mg	6.20 mg	<0.007	<0.007	0.12 ± 0.03	0.22 ± 0.07*	0.22 ± 0.10	0.40 ± 0.09*	0.05 ± 0.03	0.07 ± 0.05
22:6 (n-3) docosahexaenoic acid	<0.24 mg	69.44 mg	<0.007	<0.007	0.56 ± 0.09	0.86 ± 0.27*	1.57 ± 0.35	3.13 ± 0.65*	9.55 ± 2.32	11.74 ± 1.06*

*Fatty acid compositional analysis of total lipids were determined, by gas chromatography—mass spectrometry (GC/MS) methods as previously described (33), in drink (n = 2/measurement), chow (30 g/measurement), plasma, liver and brain frontal cortex tissue (10 rats/group)*.

α *Rats consumed 11.9 ml (± 0.6 ml) CON/placebo drink or 12.4 ml (± 0.4 ml) AIDM/Smartfish drink per day, 5-days/week*;

# Rats consumed 23.3 g (± 1.3 g) CON/placebo chow or 21.8 g (± 1.5 g) AIDM chow per day. *Statistically different values between CON and AIDM diet groups, P < 0.05.

*CON, control drink or chow; AIDM, anti-inflammatory dietary mix; ND, not detected. Plasma and brain tissue values are mean ± SDEV for n = 10 rats/group*.

The diet consumed by the rats during the recovery period consisted of the standard AIN-76A chow (CON) or the AIDM diet (AIDM), in which the chow contained supplemental vitamin D_3_ and provided *ad libitum* (600 IU/g chow, prepared by Research Diets, Inc., New Brunswick, NJ). Additionally, to provide dietary long chain omega-3s for 5 days per week during the recovery period, CON rats consumed a 12 ml serving of a placebo juice (CON) consisting of fruit juice alone (12 kcal/serving), and AIDM rats consumed a daily 12 ml serving of AIDM juice (12 kcal/serving, Smartfish® AS, Oslo, Norway). In total, AIDM rats consumed ~65 mg of DHA and EPA per day. This is in line with the rat TBI studies of Bailes and Mills (35, 36) where DHA was provided at 40 mg/day. For the analysis of omega-3 fatty acids and vitamin D, rats (10 per group) were sacrificed after consuming the diets for 30 days. To quantify omega-3 fatty acids (DHA and EPA) levels in rat diets, plasma, liver and brain frontal cortex tissues, fatty acid compositional analysis of their methyl esters, as prepared by transesterification of extracted total lipids, were determined using gas chromatography-mass spectrometry (GC-MS) (33). Vitamin D levels in juice (200 ml/duplicate measurement) and chow (30 g/measurement) samples were determined by liquid chromatography–mass spectrometry (LC-MS) as previously described (37).

### Animal Care

Young adult 6-week-old male Wistar Han rats (Charles River Laboratories, Wilmington, MA; RGD Cat No. 38676310, RRID:RGD 38676310) were individually housed in temperature-controlled rooms with a 12-h light/dark cycle. All cages were equipped with 345 mm (diameter) voluntary running wheels (STARR Life Sciences Corp, Oakmont, PA; Cat No. Tecniplast 2154F); and rats had unrestricted access to the wheels. Each cage was also fitted with a magnetic switch to allow for the counting of wheel revolutions using VitalView Series 3000 software (STARR Life Sciences). Prior to exposure to TBI, animals were fed a commercial semi-purified AIN-76A diet *ad libitum* (Research Diets Inc., New Brunswick, NJ; Cat No. D10001) for 7–8 weeks with average body weight of 341.8 g (±29.8) attained on the day of injury. In the 30 days post-injury recovery, rats were *ad libitum* fed either the CON diet or the AIDM diet, as previously described above.

### Injury

In the Marmarou model (38), the scale of the injury is dependent on the impact height from which the mass is dropped, with the impact energy calculated from *mgh* (by multiplying mass, height, and gravitational force). Marmarou (38) dropped a 450 g brass weight from 100 cm onto a metallic helmet to produce a mild injury (=0.441 J), and a 450 g weight from 200 cm to produce a moderate injury (=0.882 J). The terms *mild* and *moderate* are taken from Marmarou (38). In our experiments, the goal was to induce a mild-to-moderate injury, within a comfortable zone of success, hence a 500 g metal slug was dropped from 120 cm (=0.588 J), which, theoretically, is between the mild- and moderate-injury models proposed by Marmarou (38). To disperse the impact area, the 500 g slug landed on a metal disc of 24.26 mm (diameter) × 1.75 mm (thickness) attached to the unshaven forehead using Velcro. To slow the rate of deceleration and to allow cranial excursion without a second impact against a hard surface, the rats were placed prone on a foam pad (Type E, Foam to Size, Inc., Ashland, VA). After the initial impact, the rat along with foam bed is pushed away in a lateral direction to prevent a secondary rebound impact. Once the average body weight exceeded 325 g, brain injury was induced by weight drop to the medial frontal cortex of the Hit rats, located centrally between lambda and bregma fissures. Prior to injury, rats were anesthetized using a cocktail of Midazolam (Dormicum, Roche Products Limited, Welwyn Garden City, UK; Cat No. 23155060142; at 2.0 mg/kg) and Medetomidine (Dexdomitor, Pfizer Animal Health, Exton, PA; Cat No. 10002752; at 0.1 mg/kg), administered subcutaneously (*s.c*.) into the nape of the neck. To minimize the effects of the anesthesia on general physiology and the pathophysiology of the injured brain, an antagonist drug combination of Antisedan (Atipamezole, Pfizer, New York, NY; Cat No. 10000449; at 5 mg/ml) and Anexate (Flumazenil, Roche, Basel, Switzerland; Cat No. 4252953; at 0.1 mg/ml) was administered *s.c*. into the flank area at 8 min post-injury. A pulse oximeter collar (STARR Life Sciences Corp) was used to monitor the heart rate (HR), breathing rate (BR) and oxygen saturation levels (O_2_ SAT) prior to, and following, head trauma to assess anesthetic depth. Body temperature was maintained using a homeothermic blanket. No Hit rats were anesthetized and subsequently treated with the antagonist drugs, but not exposed to the weight drop. All rats, including shams, recovered the righting reflex within 2–3 h of receiving the antagonist drug combination, with no evidence of seizures or paralysis at any time post-injury. We report no immediate or long-term mortality among those rats exposed to the mTBI. Body weight was measured daily throughout recovery.

### Behavioral Assessments

In our experiments, voluntary running performance was quantified as the average number of revolutions run per day (converted to km/day) at 48 h, 14 days, and 30 days following mTBI. The percent change in distance per day compared to the average daily distance run over 14 days pre-TBI was then calculated as an index of recovery. The Rotarod test is the most efficient and reliable test for vestibular motor activity in the acute post-brain injury phase (1–5 days) (39). Details for measuring evoked voluntary motor function before and after exposure to mTBI are provided in Supplementary Material. The rotating pole test is an advanced test of coordination and integration of movements (40). While the rotating pole test is based on the same principals as the Rotarod test (motor function & coordination), this test is more sensitive as it evaluates the ability of the animals to balance and to integrate and coordinate their movements to traverse a horizontal pole, rotating clockwise at 4 rpm. Further details are provided in Supplementary Material. For the Barnes Maze (BM) test, latency was scored as the “time to locate the hidden box” as previously reported (41). Details on scoring methods for the BM test used before and after exposure to mTBI, are provided in Supplementary Material.

### Tissue Preparation and Analyses

After 48 h, 14 days or 30 days on the diets following induction of mTBI, animals were deeply anesthetized and euthanized by decapitation for removal of brain tissues, liver and the collection of plasma and sera. Blood was transferred to Sarstedt monovette serum and EDTA plasma tubes and immediately placed onto ice, or until plasma was separated by centrifugation (4°C; 5 min, ~3,500 rpm). Following blood collection, brain and liver tissues were harvested and snap frozen in liquid nitrogen, and stored at −80°C until analysis. DHA and EPA concentrations of plasma, liver, and brain frontal cortex tissue were determined by GC/MS methods as previously described (33). For the analysis of neurotrauma biomarkers, an ultrasensitive single-molecule array technology (Simoa Neurology 4-plex assay, Quanterix, MA; Cat No. 102153) was used to measure T-tau, GFAP, UCH-L1, and NF-L levels in plasma. To quantify vitamin D levels in serum, a rat Vitamin D ELISA, which measures 25-hydroxyvitamin D_2_ and D_3_ (American Laboratory Products Company (ALPCO), Ltd., Salem NH; Cat No. 38-25DHU-E01), was used.

### Statistics

A power analysis indicated that *n* = 12 for the behavioral studies would give a power rating of 90% and that *n* = 6 for the biochemical studies would also allow for detection of significant differences (42–44). In the pilot study, the group consuming the AIN-76A diets (CON) was considered as the “control group” to allow for comparison to the group consuming supplemental omega-3 fatty acids and vitamin D (AIDM). In the primary study, with our interest in the effects of these diets on mTBI, the non-injured group was also labeled as a “No Hit” group for any comparisons. For the body weight gain, distance run, and the BM and rotating pole times, statistical differences between the experimental groups were tested using two-way ANOVA followed by a Bonferroni *post-hoc* test to compare treatments. All variables were examined for normality. Unpaired *t*-tests were used to evaluate the shift in phenotypic endpoints. For the rotating pole score, univariate analyses were carried out using the non-parametric Mann–Whitney U test, for comparing two groups. For plasma biomarker data, differences between groups were analyzed using general linear model (GLM) statistics (SPSS version 26.0, Chicago, IL; RRID: SCR_019096). Significance was *P* < 0.05 and all data is expressed as means ± SDEV, except for **Figure 3**, where data is expressed as means ± SEM.

## Results

Despite the administration of the anesthesia reversal drugs, rats exposed to mTBI (257.7 ± 96.4 s) took 12% longer to regain consciousness than the uninjured (No Hit) rats (230.0 ± 96.4 s, *P* = 0.3), as determined by the absence of toe and/or tail pinch reflex. All rats recovered their righting reflex abilities within 2–3 h of receiving the antagonist drug combination, with no mortality or evidence of seizures or paralysis at any time post-injury. HR, BR, and O_2_ SAT levels were recorded for 8 min pre-injury, and the monitoring continued until the rat was fully conscious. Immediately following exposure to mTBI, all rats experienced a period of significant bradycardia (11.2% decreased heart rate, *P* < 0.01), as has been previously reported in both animal models (44, 45), and in humans (46). No change in BR and thus O_2_ SAT was observed. No gross motor impairments of ambulatory ability were observed in the acute stages of recovery (0–48 h), in any of the rats, and no significant differences between Hit and No Hit rats in any of the assessment measures were observed during recovery from anesthesia.

Animals tolerated daily oral supplementation with omega-3 fatty acids and vitamin D without any observed untoward side effects. In the pilot study, we found the AIDM diet, compared to CON diet, only increased the rat brain's content of DHA by a small amount (+22.9%, *P* < 0.05), while the DHA in the corresponding plasma (+53.6%) and livers (+94%) were increased by greater amounts (*P* < 0.05, Table 1). Since the liver is the major peripheral storage site and supplier of DHA to the brain (47, 48), these changes would dramatically decrease the susceptibility of the animals to neuronal cell injuries from exposure to mTBI. In contrast, EPA levels in the frontal cortex were undetectable in both groups despite consuming ~46 mg of EPA per day, for 30 days, which is consistent with the known absence of stable EPA incorporation into brain lipids (49). EPA levels of plasma and livers, however, were markedly elevated by the AIDM diet, as hepatocytes can readily esterify this fatty acid into secreted lipids [e.g., VLDL particles (49)]. The rats on both diets maintained a constant intake of food over the course of the pilot study. Average juice consumed was 11.9 ml (±0.6 ml) of the CON juice per rat/day, or 12.4 ml (±0.4 ml) of the AIDM juice per rat/day. Average chow consumed was 23.3 g (±1.3 g) of the CON diet per rat/day, or 21.8 g (±1.5 g) of the AIDM diet per rat/day (Table 1).

**Figure 2:**
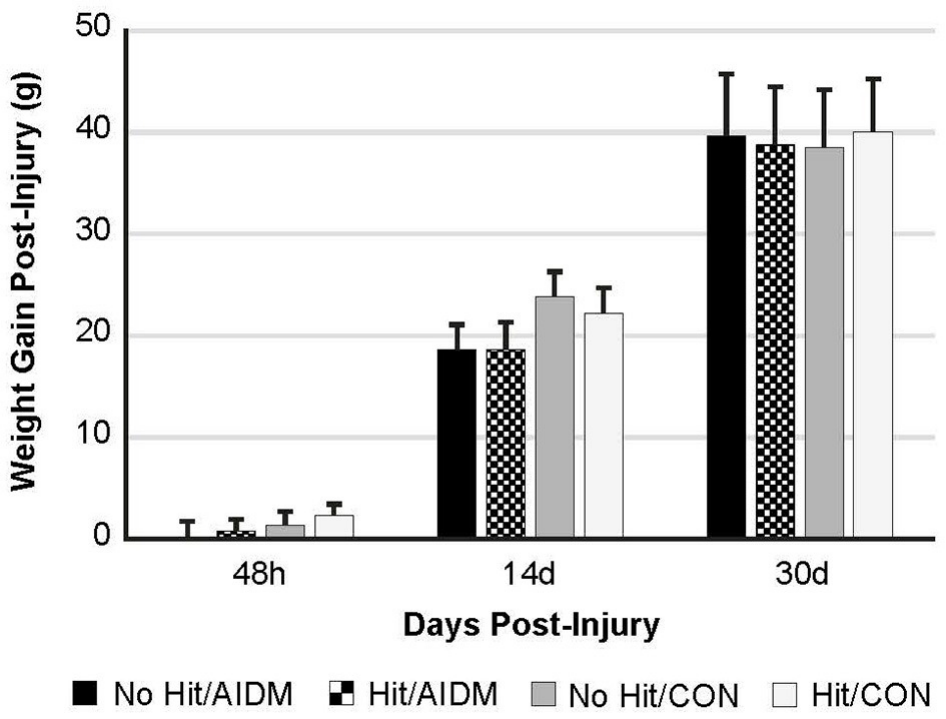
Weight gain (grams) at 48 h, 14 days, and 30 days following mTBI in rats consuming either anti-inflammatory (AIDM) or control (CON) diets. Rats were divided into TBI-exposed (Hit) or non-TBI (No Hit) control groups. Body weight was measured pre-injury and assigned a baseline zero value. Weight gain was measured just prior to euthanasia for tissue harvesting. There is no significant difference between groups at any of the three end-points of 48 h, 14 days, and 30 days. Bar heights represent mean value ± SDEV for *n* = 30 rats/group at 48 h, and *n* = 24 rats/group at 14 days, and *n* = 12 rats/group at 30 days.

In the primary study, intakes of juice and chow were not different from the pilot study. In addition, consistent with the food intake, and with similar energy levels in the juices (12 kcal/serving), all of the rats showed a comparable gain in weight 39.25 g (±0.70 g) over the 30 days of post-TBI recovery, independent of diet or injury status (Figure 2). In rats consuming the AIDM diets for 30 days post-injury, the serum levels of 25-hydroxyvitamin D were significantly higher than in rats fed the CON diets (+37.3%, *P* < 0.05, Table 2), with no effect of injury.

**Table 2:** Vitamin D consumption, and resultant serum values after 30 days on CON or AIDM diets post-TBI.

**Ingredient**	**CON drink consumed/day^**α**^**	**AIDM drink consumed/day^**α**^**	**CON chow consumed/day^**#**^**	**AIDM chow consumed/day^**#**^**	**CON rat serum (ng/mL)**	**AIDM rat serum (ng/mL)**
Vitamin D_2_	<0.25 IU	<0.06 IU	<4.0 IU	<4.0 IU	128.2 ± 3.4	176.0 ± 14.5*
Vitamin D_3_	<0.50 IU	513.77 IU	17.6 IU	160.4 IU*		

*Vitamin D levels in drink (n = 2/measurement) and chow (30 g/measurement) samples were determined by liquid chromatography–mass spectrometry (LC-MS) methods as previously described (37). Vitamin D levels in sera were determined using a rat Vitamin D ELISA (ALPCO, Ltd.), which measures 25-hydroxyvitamin D_2_ and D_3_*.

α Rats consumed 11.9 ml (± 0.6 ml) CON/placebo drink or 12.4 ml (± 0.4 ml) AIDM/Smartfish drink per day, 5-days/week; ^#^Rats consumed 23.3 g (± 1.3 g) CON/placebo chow or 21.8 g (± 1.5 g) AIDM chow per day. *Statistically different values between CON and AIDM diet groups, P < 0.05.

*CON, control drink or chow; AIDM, anti-inflammatory dietary mix. Serum values are mean ± SDEV for n = 11–12 rats/group*.

**Figure 3:**
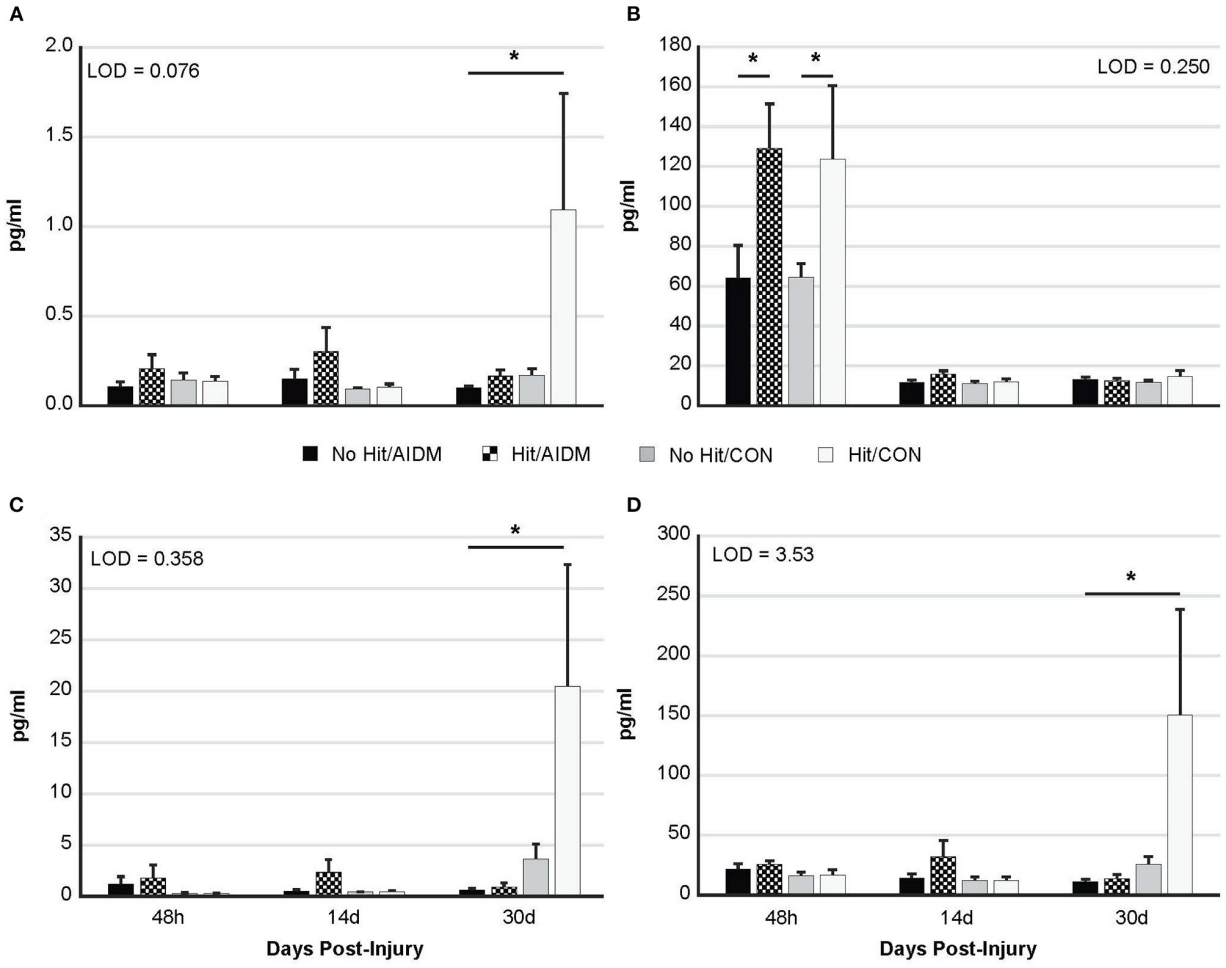
Plasma levels (pg/ml) of four neurotrauma biomarkers at three end-points post-injury of 48 h, 14 days, and 30 days. Rats consumed either anti-inflammatory (AIDM) or control (CON) diets for up to 30 days, and were divided into TBI-exposed (Hit) or non-TBI control (No Hit) groups. **(A)** T-tau, **(B)** NF-Light, **(C)** GFAP, and **(D)** UCH-L1. T-tau, total tau; NF-L, neurofilament-L; GFAP, Glial Fibrillary Acidic Protein; UCH-L1, Ubiquitin Carboxyl-terminal Hydrolase L1; LOD, Limit of Detection (pg/ml). *Groups that significantly differ from relevant non-injured groups, *P* < 0.005; Bar heights represent mean value ± SEM for *n* = 6 rats/group at 48 h, and *n* = 12 rats/group at 14 and 30 days.

Increased plasma NF-L levels post-injury reflects the release of proteins from the breakdown of neurofilaments into the cerebrospinal fluid (CSF) following axonal damage within the brain as a result of the injury. At 48 h post-injury there was a significant mTBI-induced increase in plasma levels of NF-L (97.5%, *P* < 0.005), with no effect of diet (Figure 3B). In rats consuming AIDM diets for 30 days, plasma T-tau, GFAP, and UCH-L1 levels in rats exposed to TBI (Hit) were not different from Control (No Hit) rats (Figure 3), whereas in rats consuming the CON diets, plasma T-tau, GFAP, and UCH-L1 levels were significantly higher (average 496.5%, *P* < 0.005) in the Hit group, compared to rats in the No Hit group.

**Figure 4:**
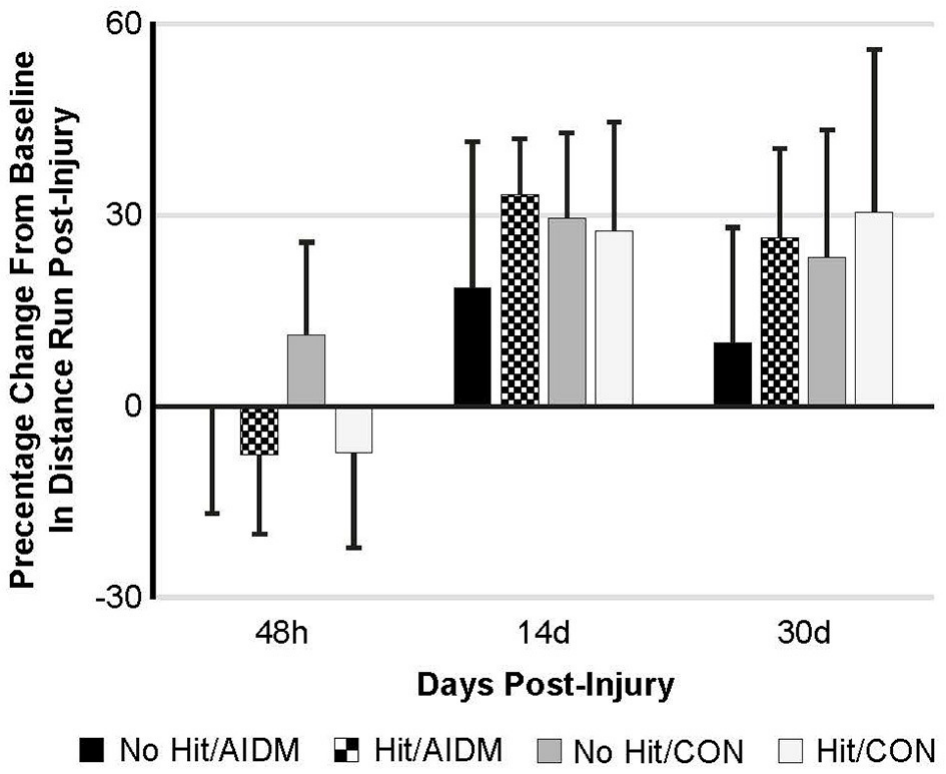
Percentage change from baseline in distance run post-injury at 48 h, 14 days, and 30 days following mTBI in rats consuming either anti-inflammatory (AIDM) or control (CON) diets (onset of injury assigned as Day 0). Rats were divided into TBI-exposed (Hit) or non-TBI (No Hit) control groups. Average distance run over 14 days pre-TBI was measured and assigned a baseline zero value. For each of the time points, values are from animals that experienced 30 days of recovery. There is no significant difference between animals at any of the three time points. Bar heights represent mean value ± SDEV, *n* = 11–12 rats/group.

**Figure 5:**
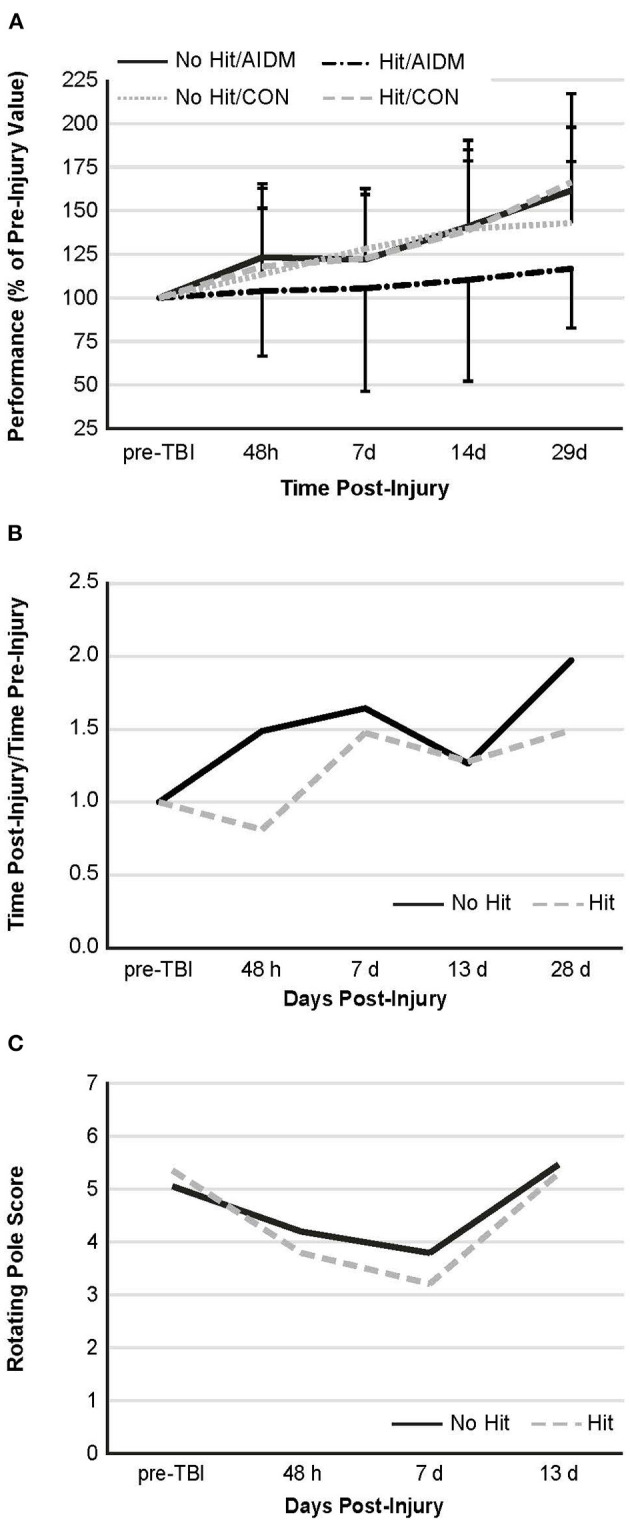
Behavioral measures. **(A)** Rotarod performance—mean duration (latency to fall time) on the Rotarod at four time points post-injury, as a percentage of the time sustained on the apparatus pre-injury. There is no significant difference between groups at any of the time points post-injury. Values are mean ± SDEV, for 11–12 rats/group B and C. Rotating pole performance—mean effort to traverse the pole post-TBI, as compared to that required on the apparatus pre-injury. Rats were divided into TBI-exposed (Hit) or non-TBI (No Hit) control groups. Performance was scored as mobility time to traverse the pole, as normalized to that measured pre-TBI **(B)** or using a composite score on a rank scale of 0–6, accounting for duration, foot slips, and distance traveled **(C)**. Pre-injury scores were determined at two days before exposure to TBI, with all animals undergoing training until they achieved a score of 5 or 6. With no dietary effects being present, rats were then grouped by injury. There are no significant differences between animals at any of the time points post-injury. As these are non-parametric data, the values shown are representative of means alone, with *n* = 11–12 rats/group. There is no significant difference, as judged by the Mann-Whitney U test, between animals at any of the time points post-injury.

**Figure 6:**
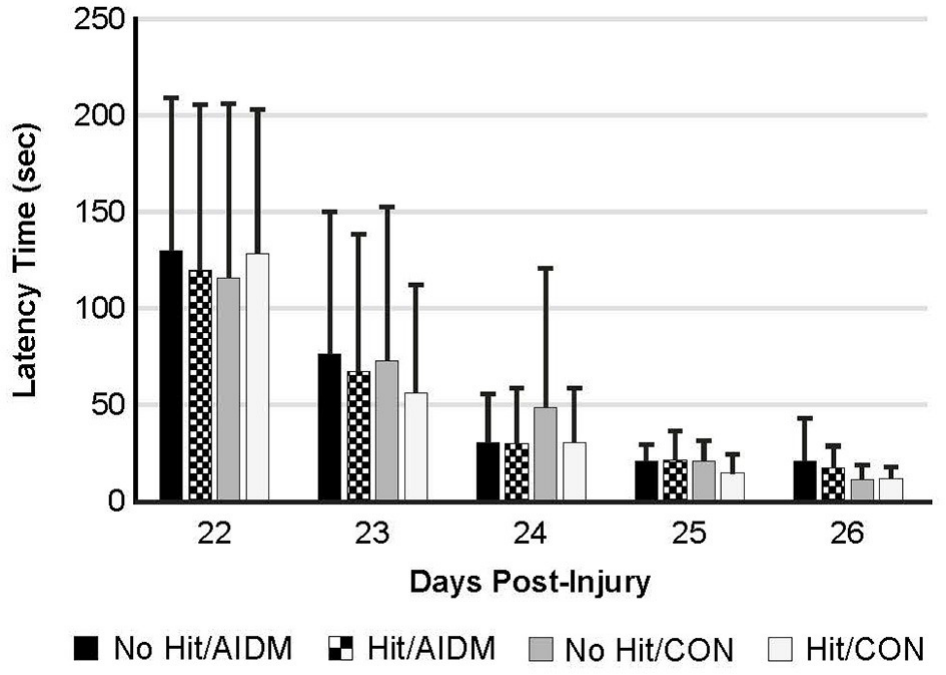
The Barnes Maze was used to determine spatial learning and memory retention in TBI-exposed (Hit) and non-TBI (No Hit) rats. Pre-injury, cumulative average latency (sec) to locate the hidden box was measured over 5 days (days−8 to−4, with date of injury assigned day 0). The anti-inflammatory (AIDM) or control (CON) diets were started on Day 0. On days 22–26 post-TBI, with the box moved to a new location (~90 degrees away from the first location), latency was again measured over 5 consecutive days and values reported at each. No significant difference was found between animals at any time points. Bar heights represent mean value ± SDEV, for *n* = 12 rats/group.

Behavioral tests conducted did not demonstrate injury-effects despite the significant increase in plasma NF-L levels at 48 h post-injury (*P* < 0.005). Similarly, there were no apparent dietary-effects on behavioral measures despite the increased plasma (53.6%) and increased brain frontal cortex (22.9%) levels of DHA. Neither the mTBI nor the AIDM-supplemented diet had any impact on voluntary running capacity (Figure 4), the RotaRod (Figure 5A), or the rotating pole test (Figures 5B,C), at any time during the 30 days recovery period. Rats displayed consistent circadian rhythms during the pre-injury time, running mostly at night (89.6 ± 2.1%), and at the end of the 30 days recovery, the amount of running at night was not different from pre-injury. Rats ran on average 2.93 ± 0.88 km daily during the 30 days recovery, and statistical analysis revealed no difference between Hit and No Hit animals in daily running in the 48 h post-injury period, and there was no difference in daily exercise between 48 h and 30 days, post-injury. Neither the mTBI, nor the post-injury change in diet, had any impact on RotaRod performance measured at 48 h, 7 days, 14 days, and 29 days post-injury (Figure 5A). Similarly on the rotating pole test, neither the mTBI, nor the post-injury change in diet, had any effect on rotating pole performance (Figure 5B) or when scored on a rank scale of 0–6, accounting for duration, foot slips, and distance traveled (Figure 5C) measured post-injury. Overall performance in the BM improved across time for all groups, as indicated by a decrease in latency across 5 days, but there were no significant differences between groups due to injury or diet (Figure 6).

## Discussion

The present study used a rat model of mTBI to evaluate the effect of an anti-inflammatory diet, as supplemented with omega-3 fatty acids and vitamin D_3_, on four neurotrauma biomarkers (T-tau, GFAP, UCH-L1, and NF-L) and on neuro-behavioral outcomes. Because of the complex and multifactorial nature of TBI, we hypothesized that a combination of these two putative beneficial dietary components would have synergistic value in reducing the neuroinflammation and behavioral deficits associated with mTBI. Our findings indicate that the post-TBI administration of a combination of omega-3 fatty acids (DHA and EPA) and vitamin D_3_ for 30 days significantly decreased plasma levels of three of the biomarkers indicating the presence of neurotrauma, namely T-tau, GFAP, and UCH-L1 to pre-injury levels (Figure 3). These findings greatly outweigh the apparent lack of omega-3s and vitamin D_3_ toward modulating behavioral outcomes in the animals. Therefore, omega-3 fatty acids and vitamin D supplementation are suitable therapeutic candidates against mechanical insult-induced trauma to the brain.

Previous work has shown a significant, strong correlation between T-tau, GFAP, and UCH-L1 and the extent and severity of neuronal cell damage in patients with TBI (19). Therefore, our observation that rats consuming AIDM diets exhibited at least a reduction of plasma T-tau levels to non-injured levels 30 days post-TBI is an important new development. Tau is a cytoskeletal protein involved in stabilization of axonal microtubules, which following brain injury is upregulated in expression. Tau elevation is observed in the cerebrospinal fluid (CSF) of patients with neurodegenerative disease and head injuries, suggesting its extracellular release during neuronal damage and a role as a biomarker with specificity for TBI. Repeated concussions can also stimulate hyperphosphorylation of tau, which leads to the chronic formation of neurofibrillary tangle aggregates. Thus, tau is also a predictor of delayed recovery after mTBI (50). For example, in a study where plasma and serum levels of T-tau were monitored, using an electrochemiluminescence-based sandwich immunoassay, in ice hockey players prior to and after concussions (51), the concentration of T-tau 1-h after concussion correlated with the number of days it took for concussion symptoms to resolve (ρ = 0.60; 95% CI, 0.23–0.90; *P* = 0.002). The timeline for the increased T-tau in the present study is not unlike what others have reported. A recent study similarly used an ultrasensitive immunoassay technology (EIMAF) to document changes in T-tau and phosphorylated tau (P-tau) proteins in two rodent models following severe TBI (52). They reported that serum T-tau and P-tau levels generally increased during the acute stage of severe TBI in rats (from Days 2 to 30 for T-tau), and that during the subacute/chronic state (Days 14–30), the increased levels of both T-tau and P-tau were maintained in these animals. Therefore, interventions, such as the AIDM diet investigated in our study, that reduce T-tau following TBI, could prevent excitotoxin-induced abnormalities in neuronal activity associated with TBI (53–55), and potentially mitigate the disruption of specific cognitive functions.

Our initial research objective focused on improving some degree of behavioral outcome through diet, regardless of how modest. It is well-documented that the Marmarou model used in our study produces a diffuse axonal injury typically associated with falls and motor vehicle accidents (56). The brain injury was induced to the medial frontal cortex, which is implicated in a number of basic and complex behaviors, and we tested a diverse range of behavioral procedures in our study. Surprisingly, the injury had no apparent effects on five different measures of behavior: voluntary running, RotaRod, Rotating Pole, and Barnes Maze. The lack of behavioral or cognitive deficits in our model is not unique to the current study. In a recent review of behavioral measures used to assess weight drop models, only 45% of studies using the Rotarod assay report a TBI-induced deficit (57). It is possible that the magnitude of injury induced by the technique employed in this study was not sufficient to produce a discernable deficit in behavior using this test battery. Alternatively, some other effect of our experimental approach may have mitigated behavioral deficits. For example, too many behavioral tests might have been done on the same animals; if so, that approach might have impacted their performance abilities. We noted that when rats were trained on both the Rotarod and rotary pole tests on the same day, they readily become confused between the two rotational directions, i.e., parallel vs. perpendicular running, respectively.

Our rats are always preferentially housed in cages fitted with voluntary running wheels as a form of enrichment activity, so the initial premise of examining the beneficial effects of an anti-inflammatory diet on behavior, in effect, became a combination study of exercise and dietary interventions post-TBI. While we did not incorporate rats without running wheels into our study as non-exercise controls, a number of other reported studies have established that voluntary exercise facilitates recovery after TBI in rats, with improvements in depression, vestibular motor activity, anxiety, and memory & learning (58–62). However, these earlier studies did not report on the effects of voluntary running on neurotrauma biomarkers post-TBI. In the current study, in rats consuming standardized (CON) chow and exposed to TBI, plasma levels of T-tau, GFAP and UCH-L1 were significantly elevated above non-injured control rats at 30 days (*P* < 0.005; Figure 3), indicating the behavioral benefits of voluntary exercise had not transferred to these pathological indicators of neurotrauma. In fact, the lack of behavioral deficits in the mTBI (Hit) control diet rats could in part represent neuroprotective benefits from the voluntary exercise alone.

The reduction of plasma GFAP and UCH-L1 at 30 days post-TBI in rats consuming AIDM diets indicate different cellular responses to the AIDM diet. While GFAP is an astroglial (cytoskeletal protein) biomarker of injury, UCH-L1 is a neuron-specific brain injury biomarker, related to enzymatic degradation of damaged proteins via the ubiquitination pathway, so changes in UCH-L1 essentially reflect the health status of the entire brain. Thus, a significant decrease in plasma levels of GFAP and UCH-L1 reflect deactivation of glial cells, and recovery of neurons, respectively. Several studies have shown that both the predictive value of UCH-L1 and its correlation with injury severity increase substantially when combined with GFAP (63, 64).

Neurofilament light chain (NF-L) protein is an abundantly expressed cytoskeletal component of large caliber, myelinated subcortical axons contributing to the strength of the axon (65), and is released into the CSF and then blood following axonal injury or degeneration (66, 67). Acutely, we observed significantly increased plasma NF-L levels (*P* < 0.005; Figure 3B), but not in T-tau, GFAP, or UCH-L1, as has been reported by others (68, 69). Plasma NF-L levels better reflect *acute* exposure whereas plasma T-tau levels signify *chronic* change in brain structure over time (70). Despite excellent rodent cross-reactivity of the ultrasensitive Simoa NF-L assay, relatively few preclinical studies have utilized this assay in the context of TBI assessments (71–73). Numerous studies have found serum NF-L to be a dynamic biomarker of axonal pathology neurodegenerative diseases (74), with diffuse axonal injury hypothesized to be a key component of symptom outcomes following TBI (75, 76). For example, following concussion, serum NF-L was increased in professional ice hockey players compared to non-injured controls, and changes in NF-L levels followed an upward trajectory with the highest concentrations seen at 12- and 144-h post-injury (77). As serum levels of NF-L reportedly peak at 6 h after both injury and anesthesia in mice (78), the decrease in NF-L to background levels at 14 days and 30 days was expected; and was not likely influenced by the AIDM diet. Unlike plasma T-tau, serum NF-L concentration is not affected by physical exertion (67), which is crucial since our rats were individually housed in cages fitted with voluntary running wheels throughout the study. Our rats were also all exposed to the same anesthesia drugs, which has previously been reported to acutely increase NF-L levels in both serum and CSF (78).

Previous research has demonstrated neuroprotective efficacy of a number of nutrients. For example, the neuroprotective role of vitamin D monotherapy in TBI has been reported in preclinical studies, but in those studies, vitamin D was administered intraperitoneally in combination with progesterone (31, 32) as a single bolus of 5 μg/kg at 1 h post-injury, making it unclear if there was a synergistic steroidal hormone effect on enhancing recovery. In the present study, rats consumed diets supplemented with 4.0 mg vitamin D_3_/day for 30 days, and serum levels of vitamin D increased by 37.3% (*P* < 0.05), compared to rats consuming the CON diets. The beneficial effects on markers of neurotrauma that we observed are consistent with findings of a recent study by Sharma and colleagues that evaluated the effects of a one-time oral dose of 3.0 mg vitamin D, and on clinical outcomes showed a strongly beneficial effect of vitamin D supplementation with improvement of the Glasgow Coma Scale (GCS), and a shortening of the mechanical ventilation period in these patients.

The present study combined dietary vitamin D_3_ supplementation with long chain omega-3 fatty acids to reduce post-TBI neuroinflammation. In this study, the CON diets had negligible EPA and DHA content, while the AIDM diets provided a daily average of 46 mg EPA and 69 mg DHA, resulting in significantly increased DHA levels in plasma, liver and brain tissues (Table 1). However, the effects of the AIDM diet on EPA levels were not as clear. After 30 days on the supplemented diets, plasma levels of EPA increased from not detectable, to 1.25% of total PUFAs; but the supplementary dietary EPA did not accumulate in the brain frontal cortex tissue, and instead is eliminated by oxidation, or converted to some DHA within the astrocytes or liver hepatocytes (79). In contrast, plasma levels of DHA increased significantly (53.6%, *P* < 0.05) in rats consuming AIDM diets, and DHA levels in the frontal cortex increased by 22.9% (*P* < 0.05), from 9.55 to 11.74% of total PUFAs. Previous reports indicate that DHA contributes to clearance of amyloid-β in the brain (80, 81), and both DHA and DHA-derived metabolites such as neuroprotectin D1 (82) and resolvins (83) protect against neurodegeneration. Likewise, EPA can directly inhibit prostaglandin synthesis and be converted to inflammation resolving metabolites, e.g., resolvin E1, but both having a lower bioactivity than that of DHA (84).

The current animal study was designed to “treat” TBI-related symptoms, including depression and/or anxiety with a combination of EPA and DHA, and the AIDM juice provided post-injury contained equal amounts of EPA:DHA, with the manufacturer also willing to provide a placebo juice without the supplemental omega-3 and vitamin D. However, with minimal uptake of EPA into neuronal tissues (Table 1), the significantly altered profiles of plasma T-tau, GFAP and UCH-L1 post-TBI cannot be attributed to the dietary EPA in this model. This is contrary to what has previously been reported by Bauer et al. (85) in a recent clinical trial: their findings suggest EPA-rich supplementation may be more beneficial in improving behavioral cognitive outcomes compared to DHA-rich supplementation in healthy populations. Bauer et al. (85) compared a high EPA intervention group (3:1 ratio EPA:DHA) and a high DHA intervention group (4:1 ratio DHA:EPA) supplemented for 30 days. Behavioral outcomes were significant in the EPA-rich group; reaction times were decreased compared with supplementation rich in DHA (*P* = 0.04) whereas DHA-rich supplementation did not induce any behavioral improvement. It was concluded that following the 30 day intervention period EPA-rich supplementation was more successful than DHA-rich supplementation in improving neural efficiency during higher order cognitive tasks.

Considering that the vast difference for efficacy of EPA alone in ameliorating depression and anxiety versus not, for TBI, lies in the underlying mechanisms that it is targeting. For depression and anxiety, EPA and DHA are modulating gene expression and activity of dopamine and serotonin receptors, where they are both known to directly activate various transcription factors (e.g., AKT phosphorylation). This in turn helps restore the neurotransmitter imbalances associated with these psychiatric disorders. However, EPA appears to be a much stronger ligand than DHA for the associated transcription factors. In contrast, for alleviating TBI and spinal cord injuries (i.e., neuronal cell damage), DHA is more efficacious than EPA, since it is suppressing immune cell and enzyme activities involved in neuroinflammation processes (e.g., neutrophil migration and COX2 activity). In this case, it is well known that DHA and its metabolites (e.g., neuroprotectin D1) have a much higher binding affinity/potency than EPA and related molecules (e.g., resolvin E1). This may also explain the significant uptake of DHA from the AIDM into neuronal tissue observed in the current study, which functions in the phospholipids of neuronal cell membranes to establish lipid bilayer fluidity and thus signal transduction protein mobility.

Finally, the inclusion of EPA is an unavoidable fact of using fish oil as the supplement source, as it is always present in marine fish tissues/fat in a ratio to DHA of 1:1 – 2:1, due to the original algae to krill source of these two fatty acids in the food chain. There are commercially available oils made from “customized” farmed algae strains (e.g., *Crypthecodinium cohnii*) that contain only DHA (e.g., DHASCO made by Martek Biosciences, DSM Nutritional Products), but these should be considered as largely artificial/man-made dietary sources of long chain polyunsaturated fatty acids (LCPUFAs). In essence, EPA is likely not essential for our findings, but it is also an unavoidable fact of the dietary supplement that was used. Thus, our approach has a good base justification.

TBI often causes a transient loss of appetite and decreased BW after the injury (86), and the consumption of dietary omega-3 fatty acids reportedly suppresses appetites and upregulates expression of genes involved in energy metabolism (87). However, we did not see any decrease in BW in any groups over the 30 days of recovery (Figure 2). In fact, the rats gained weight, with the gain over 30 days of recovery being similar between groups (Figure 2), and apparently not influenced by the presence of the omega-3 fatty acids in the AIDM chow, or the induction of mTBI. However, the rate of gain in the current study (1.3 g/d) was much less than the 5.8–6.3 g/d that others have reported in studies in which rats were provided a diet supplemented with DHA before and after a controlled cortical impact (CCI) injury. In that study, rats were not exercised, and likely maintained a much more positive energy balance (88). Regardless, similar to our findings, the weight gain observed after injury in that study, was not different between the injured and sham groups (88). Thus, our findings indicate that exercise appears to provide support to pathways that preserve cognitive function following mTBI, and exercise may have the added therapeutic effect of reducing weight gain post-injury, as has been reported (89).

## Strengths and Limitations

A strength of our study is the use of ultrasensitive single molecule immunoassay array (Simoa) technology. T-tau concentrations in the peripheral blood, in particular, are very low and almost impossible to measure precisely by most of the conventional immunoassays available (90). The Simoa technology (Quanterix) is sensitive enough to reach single-molecule detection (SMD) capabilities and has recently validated for clinical use in the diagnosis of TBI, as well as identifying different types of cellular injury (21).

The Marmarou model replicates a high-impact closed head trauma event with acceleration–deceleration forces, leading to stretching and deformation of the brain tissue, resulting in diffuse axonal injury, typically associated with falls and motor vehicle accidents (91). Additional rat TBI paradigms, such as CCI, lateral fluid percussion injury (LFPI), and/or blast over-pressure (BOP) wave injury will be required to generalize our findings. This study only examined effect(s) of brain injury or diet on four biomarkers in plasma—others should be considered (e.g., SB100) as our findings may not entirely reflect the neuropathophysiological processes in the brain following head trauma. Biochemical biomarker studies on brain tissues/brain regions are necessary to assess qualitative and quantitative similarities and/or differences between plasma and neuronal indices. In addition, neuropathological studies utilizing immunohistochemistry would aid in identifying the brain regions of biomarker localization in untreated and AIDM-treated rats subjected to mTBI. The detection of neurotrauma markers in the CSF or directly in the brain tissue is more specific as well as reliable for detecting the injuries in animal models as compared to blood, due to potential systemic influences (92). While the long-term longitudinal sampling conducted here allows for better evaluation of time-dependent changes in plasma biomarkers covering the injury- (*acute*) and dietary-response (*subacute*) phases of TBI, further work is required to establish additional injury-related neuropathologies that result from the injury, including additional markers of axonal degeneration, demyelination, gliosis, etc.

In line with the present study, there have been reports of focal brain injuries in rodents, as produced by weight drop concussion or CCI exposure, causing acute increases in brain vasopressin levels and thus elevated edema, activated astrocytes, and loss of axons (93–96). Some of these studies (94, 95), showed that giving the animals antagonist drugs toward vasopressin receptors (V1a and V2) can prevent all of these neuropathological changes post-TBI. Due to potential systemic stress in animals wounded by exposure to mTBI, there could be a subsequent increase in vasopressin release from the hypothalamus, even though this region was likely not directly perturbed. The force imparted to the brain by the weight drop concussion method was mostly limited to disrupting surface areas including the upper cortical neuron layers (gray matter) and associated corpus callosum white matter tracts. Regardless, release of vasopressin could lead to impaired cerebral blood flow (e.g., hypertension) as well as associated edema from leaking damaged vessels at the weight drop impact site. Thus, this would exacerbate the neurodegeneration phase following the TBI. Likewise, vasopressin is capable of directly stimulating the activation of astrocytes and associated axonal degeneration in brain gray and white matter; since astrocytes and axons of neurons, like vascular cells, can highly express surface receptors for vasopressin (V1a). For instance, in rats with a middle cerebral artery occlusion (MCAO), even though no histological damage is identifiable in the hypothalamus, plasma vasopressin levels are increased significantly (97). Importantly, this was not restricted to negative effects on cerebral blood vessels, since blocking activation of vasopressin responsive neurons in the supra-optic nucleus alleviates MCAO-evoked abnormal astrocytic plasticity and brain injury (97, 98). Of course, we would also need to measure the plasma or brain levels of vasopressin in our weight drop TBI-exposed animals to determine if this is potentially contributing to the observed increases in the blood biomarkers of neuronal injury.

## Recommendations

Given the failures in clinical translation of drug therapies to TBI, rigorous preclinical approaches to therapeutic screening of select nutrients, or a combination thereof, may be important for the ultimate translation of therapies to the human condition. While current treatments include surgical approaches [including decompressive craniectomy (99)] and prolonged sedation (100), there is currently no effective pharmacological treatment available for mitigating secondary injury after acute TBI and preventing the later development of dementia, Alzheimer's disease or chronic traumatic encephalopathy (101, 102). While albeit intuitive, multi-or-combinational therapeutic strategies have historically received only modest attention. Therapies that have been shown to produce beneficial effects on their own and that may provide additive benefit when combined, or that have modest effects on their own but act synergistically to exert positive outcomes, should be strongly pursued. Consistent with this, our findings suggest that a combination of omega-3 fatty acids and vitamin D could serve as easily accessible and inexpensive dietary supplements that can combat neuroinflammation and promote brain tissue repair after mTBI. The diet components used in this study can be orally administered (in addition to the parenteral route), and are readily taken up by the brain. Ultimately, this information could be used to develop a therapy to alleviate symptoms brought on by TBI in civilian as well as military populations.

## Data Availability Statement

The raw data supporting the conclusions of this article will be made available by the authors, without undue reservation.

## Ethics Statement

The animal study was reviewed and approved by Institutional Animal Care and Use Committee (IACUC), US Army Research Institute of Environmental Medicine, Natick, MA 01760-5007, United States.

## Author Contributions

AS and MC contributed to conception and design of the study. AL organized the database. MC performed the statistical analysis. AS wrote the first draft of the manuscript. AS, MC, and JD all wrote sections of the manuscript. All authors contributed to manuscript revision, read, and approved the submitted version.

## Conflict of Interest

The authors declare that the research was conducted in the absence of any commercial or financial relationships that could be construed as a potential conflict of interest.

## Abbreviations

AIDM: anti-inflammatory dietary mix
BM: Barnes Maze
CSF: cerebrospinal fluid
DHA: docosahexaenoic acid
EPA: eicosapentaenoic acid
GFAP: glial fibrillary acidic protein
NF-L: neurofilament light chain
TBI: traumatic brain injury
T-tau: total tau
UCH-L1: ubiquitin carboxy-terminal hydrolase L1.

## References

[1] Dewan MC Rattani A Gupta S Baticulon RE Hung YC Punchak M. Estimating the global incidence of traumatic brain injury. J Neurosurg. (2019) 130:1080–97. 10.3171/2017.10.JNS17352 29701556

[2] Maas AIR Menon DK Adelson PD Andelic N Bell MJ Belli A. Traumatic brain injury: integrated approaches to improve prevention, clinical care, and research. Lancet Neurol. (2017) 16:987–1048. 10.1016/S1474-4422(17)30371-X 29122524

[3] James SL Theadom A Ellenbogen RG Bannick MS Montjoy-Venning W Lucchesi LR. Global, regional, and national burden of traumatic brain injury and spinal cord injury, 1990-2016: a systematic analysis for the global burden of disease study 2016. Lancet Neurol. (2019) 18:56–87. 10.1016/S1474-4422(18)30415-0 30497965 PMC6291456

[4] DeWitt DS Hawkins BE Dixon CE Kochanek PM Armstead W Bass CR. Pre-clinical testing of therapies for traumatic brain injury. J Neurotrauma. (2018) 35:2737–54. 10.1089/neu.2018.5778 29756522 PMC8349722

[5] Bragge P Synnot A Maas AI Menon DK Cooper DJ Rosenfeld JV. A state-of-the-science overview of randomized controlled trials evaluating acute management of moderate-to-severe traumatic brain injury. J Neurotrauma. (2016) 33:1461–78. 10.1089/neu.2015.4233 26711675 PMC5003006

[6] Kline AE Leary JB Radabaugh HL Cheng JP Bondi CO. Combination therapies for neurobehavioral and cognitive recovery after experimental traumatic brain injury: is more better? Prog Neurobiol. (2016) 142:45–67. 10.1016/j.pneurobio.2016.05.002 27166858 PMC4914431

[7] Kochanek PM Bramlett HM Shear DA Dixon CE Mondello S Dietrich WD. Synthesis of findings, current investigations, and future directions: operation brain trauma therapy. J Neurotrauma. (2016) 33:606–14. 10.1089/neu.2015.4133 26671284

[8] Mayer AR Quinn DK Master CL. The spectrum of mild traumatic brain injury: a review. Neurology. (2017) 89:623–32. 10.1212/WNL.0000000000004214 28701496 PMC5562956

[9] Wilde MC Castriotta RJ Lai JM Atanasov S Masel BE Kuna ST. Cognitive impairment in patients with traumatic brain injury and obstructive sleep apnea. Arch Phys Med Rehabil. (2007) 88:1284–8. 10.1016/j.apmr.2007.07.012 17908570

[10] Hamm RJ Dixon CE Gbadebo DM Singha AK Jenkins LW Lyeth BG. Cognitive deficits following traumatic brain injury produced by controlled cortical impact. J Neurotrauma. (1992) 9:11–20. 10.1089/neu.1992.9.11 1619672

[11] Fujimoto ST Longhi L Saatman KE Conte V Stocchetti N McIntosh TK. Motor and cognitive function evaluation following experimental traumatic brain injury. Neurosci Biobehav Rev. (2004) 28:365–78. 10.1016/j.neubiorev.2004.06.002 15341032

[12] Hicks RR Smith DH Lowenstein DH Saint Marie R McIntosh TK. Mild experimental brain injury in the rat induces cognitive deficits associated with regional neuronal loss in the hippocampus. J Neurotrauma. (1993) 10:405–14. 10.1089/neu.1993.10.405 8145264

[13] Broglio SP Puetz TW. The effect of sport concussion on neurocognitive function, self-report symptoms and postural control : a meta-analysis. Sports Med. (2008) 38:53–67. 10.2165/00007256-200838010-00005 18081367

[14] Dougan BK Horswill MS Geffen GM. Athletes' age, sex, and years of education moderate the acute neuropsychological impact of sports-related concussion: a meta-analysis. J Int Neuropsychol Soc. (2014) 20:64–80. 10.1017/S1355617712001464 23375058

[15] Williams RM Puetz TW Giza CC Broglio SP. Concussion recovery time among high school and collegiate athletes: a systematic review and meta-analysis. Sports Med. (2015) 45:893–903. 10.1007/s40279-015-0325-8 25820456 PMC4441834

[16] Rohling ML Binder LM Demakis GJ Larrabee GJ Ploetz DM Langhinrichsen-Rohling J. A meta-analysis of neuropsychological outcome after mild traumatic brain injury: re-analyses and reconsiderations of Binder et al. (1997), Frencham et al. (2005), and Pertab et al. (2009). Clin Neuropsychol. (2011) 25:608–23. 10.1080/13854046.2011.565076 21512956

[17] Quinn DK Mayer AR Master CL Fann JR. Prolonged postconcussive symptoms. Am J Psychiatry. (2018) 175:103–11. 10.1176/appi.ajp.2017.17020235 29385828 PMC6586466

[18] Lange RT Brickell TA Ivins B Vanderploeg RD French LM. Variable, not always persistent, postconcussion symptoms after mild TBI in U.S. military service members: a five-year cross-sectional outcome study. J Neurotrauma. (2013) 30:958–69. 10.1089/neu.2012.2743 23205671

[19] Thelin E Al Nimer F Frostell A Zetterberg H Blennow K Nystrom H. A serum protein biomarker panel improves outcome prediction in human traumatic brain injury. J Neurotrauma. (2019) 36:2850–62. 10.1089/neu.2019.6375 31072225 PMC6761606

[20] Lewis LM Schloemann DT Papa L Fucetola RP Bazarian J Lindburg M. Utility of serum biomarkers in the diagnosis and stratification of mild traumatic brain injury. Acad Emerg Med. (2017) 24:710–20. 10.1111/acem.13174 28170122

[21] Korley FK Yue JK Wilson DH Hrusovsky K Diaz-Arrastia R Ferguson AR. Performance evaluation of a multiplex assay for simultaneous detection of four clinically relevant traumatic brain injury biomarkers. J Neurotrauma. (2018) 36:182–7. 10.1089/neu.2017.5623 29690824 PMC6306681

[22] Bazarian JJ Biberthaler P Welch RD Lewis LM Barzo P Bogner-Flatz V. Serum GFAP and UCH-L1 for prediction of absence of intracranial injuries on head CT (ALERT-TBI): a multicentre observational study. Lancet Neurol. (2018) 17:782–9. 10.1016/S1474-4422(18)30231-X 30054151

[23] McCrea M Broglio SP McAllister TW Gill J Giza CC Huber DL. Association of blood biomarkers with acute sport-related concussion in collegiate athletes: findings from the NCAA and Department of Defense CARE Consortium. JAMA Netw Open. (2020) 3:e1919771. 10.1001/jamanetworkopen.2019.19771 31977061 PMC6991302

[24] Di Pietro V Yakoub KM Caruso G Lazzarino G Signoretti S Barbey AK. Antioxidant therapies in traumatic brain injury. Antioxidants. (2020) 9:260. 10.3390/antiox9030260 32235799 PMC7139349

[25] McGeown JP Hume PA Theadom A Quarrie KL Borotkanics R. Nutritional interventions to improve neurophysiological impairments following traumatic brain injury: a systematic review. J Neurosci Res. (2021) 99:573–603. 10.1002/jnr.24746 33107071

[26] Lucke-Wold BP Logsdon AF Nguyen L Eltanahay A Turner RC Bonasso P. Supplements, nutrition, and alternative therapies for the treatment of traumatic brain injury. Nutr Neurosci. (2018) 21:79–91. 10.1080/1028415X.2016.1236174 27705610 PMC5491366

[27] Wu A Ying Z Gomez-Pinilla F. Dietary omega-3 fatty acids normalize BDNF levels, reduce oxidative damage, and counteract learning disability after traumatic brain injury in rats. J Neurotrauma. (2004) 21:1457–67. 10.1089/neu.2004.21.1457 15672635

[28] Pu H Jiang X Wei Z Hong D Hassan S Zhang W. Repetitive and prolonged omega-3 fatty acid treatment after traumatic brain injury enhances long-term tissue restoration and cognitive recovery. Cell Transplant. (2017) 26:555–69. 10.3727/096368916X693842 27938482 PMC5531869

[29] Zhu W Ding Y Kong W Li T Chen H. Docosahexaenoic acid (DHA) provides neuroprotection in traumatic brain injury models via activating Nrf2-ARE signaling. Inflammation. (2018) 41:1182–93. 10.1007/s10753-018-0765-z 29663102

[30] Hua F Reiss JI Tang H Wang J Fowler X Sayeed I. Progesterone and low-dose vitamin D hormone treatment enhances sparing of memory following traumatic brain injury. Horm Behav. (2012) 61:642–51. 10.1016/j.yhbeh.2012.02.017 22570859 PMC3517217

[31] Tang H Hua F Wang J Yousuf S Atif F Sayeed I. Progesterone and vitamin D combination therapy modulates inflammatory response after traumatic brain injury. Brain Inj. (2015) 29:1165–74. 10.3109/02699052.2015.1035330 26083048 PMC4894830

[32] Cekic M Cutler SM VanLandingham JW Stein DG. Vitamin D deficiency reduces the benefits of progesterone treatment after brain injury in aged rats. Neurobiol Aging. (2011) 32:864–74. 10.1016/j.neurobiolaging.2009.04.017 19482377 PMC3586224

[33] Rao JS Ertley RN DeMar JC Jr. Rapoport SI Bazinet RP. Dietary n-3 PUFA deprivation alters expression of enzymes of the arachidonic and docosahexaenoic acid cascades in rat frontal cortex. Mol Psychiatry. (2007) 12:151–7. 10.1038/sj.mp.4001887 16983392

[34] National Research Council. Guide for the Care and Use of Laboratory Animals. 8th ed. Washington DC: The National Academies Press (2011).

[35] Bailes JE Mills JD. Docosahexaenoic acid reduces traumatic axonal injury in a rodent head injury model. J Neurotrauma. (2010) 27:1617–24. 10.1089/neu.2009.1239 20597639

[36] Mills JD Bailes JE Sedney CL Hutchins H Sears B. Omega-3 fatty acid supplementation and reduction of traumatic axonal injury in a rodent head injury model. J Neurosurg. (2011) 114:77–84. 10.3171/2010.5.JNS08914 20635852

[37] Huang M LaLuzerne P Winters D Sullivan D. Measurement of vitamin D in foods and nutritional supplements by liquid chromatography/tandem mass spectrometry. J AOAC Int. (2009) 92:1327–35. 10.1093/jaoac/92.5.1327 19916369

[38] Marmarou A Foda MA van den Brink W Campbell J Kita H Demetriadou K. A new model of diffuse brain injury in rats. Part I: pathophysiology and biomechanics. J Neurosurg. (1994) 80:291–300. 10.3171/jns.1994.80.2.0291 8283269

[39] Hamm RJ. Neurobehavioral assessment of outcome following traumatic brain injury in rats: an evaluation of selected measures. J Neurotrauma. (2001) 18:1207–16. 10.1089/089771501317095241 11721739

[40] Mattiasson GJ Philips MF Tomasevic G Johansson BB Wieloch T McIntosh TK. The rotating pole test: evaluation of its effectiveness in assessing functional motor deficits following experimental head injury in the rat. J Neurosci Methods. (2000) 95:75–82. 10.1016/S0165-0270(99)00162-4 10776817

[41] Rosenfeld CS Ferguson SA. Barnes maze testing strategies with small and large rodent models. J Vis Exp. (2014) 84:e51194. 10.3791/51194 24637673 PMC4140524

[42] Cope EC Morris DR Scrimgeour AG VanLandingham JW Levenson CW. Zinc supplementation provides behavioral resiliency in a rat model of traumatic brain injury. Physiol Behav. (2011) 104:942–7. 10.1016/j.physbeh.2011.06.007 21699908 PMC3506179

[43] Cope EC Morris DR Scrimgeour AG Levenson CW. Use of zinc as a treatment for traumatic brain injury in the rat: effects on cognitive and behavioral outcomes. Neurorehabil Neural Repair. (2012) 26:907–13. 10.1177/1545968311435337 22331212 PMC3888640

[44] Scrimgeour AG Carrigan CT Condlin ML Urso ML van den Berg RM van Helden HPM. Dietary zinc modulates matrix metalloproteinases in traumatic brain injury. J Neurotrauma. (2018) 35:2495–506. 10.1089/neu.2017.5614 29774825

[45] dos Santos Silva D de Oliveira Brito JN Ibiapina JO Batista Lima MF de Vasconcelos Medeiros AR Carvalho e Queiroz BH. Traumatic brain injury: clinical and pathological parameters in an experimental weightdrop model. Acta Cir Bras. (2011) 26:94–100. 10.1590/S0102-86502011000200004 21445470

[46] Hackbarth RM Rzeszutko KM Sturm G Donders J Kuldanek AS Sanfilippo DJ. Survival and functional outcome in pediatric traumatic brain injury: a retrospective review and analysis of predictive factors. Crit Care Med. (2002) 30:1630–5. 10.1097/00003246-200207000-00038 12130990

[47] Igarashi M Ma K Chang L Bell JM Rapoport SI DeMar JC Jr. Low liver conversion rate of alpha-linolenic to docosahexaenoic acid in awake rats on a high-docosahexaenoate-containing diet. J Lipid Res. (2006) 47:1812–22. 10.1194/jlr.M600030-JLR200 16687661

[48] Igarashi M DeMar JC Jr. Ma K Chang L Bell JM. Upregulated liver conversion of alpha-linolenic acid to docosahexaenoic acid in rats on a 15 week n-3 PUFA-deficient diet. J Lipid Res. (2007) 48:152–64. 10.1194/jlr.M600396-JLR200 17050905

[49] Chen CT Domenichiello AF Trepanier MO Liu Z Masoodi M Bazinet RP. The low levels of eicosapentaenoic acid in rat brain phospholipids are maintained via multiple redundant mechanisms. J Lipid Res. (2013) 54:2410–22. 10.1194/jlr.M038505 23836105 PMC3735939

[50] Rubenstein R Chang B Yue JK Chiu A Winkler EA Puccio AM. Comparing plasma phospho Tau, total Tau, and phospho Tau-total Tau ratio as acute and chronic traumatic brain injury biomarkers. JAMA Neurol. (2017) 74:1063–72. 10.1001/jamaneurol.2017.0655 28738126 PMC5710183

[51] Shahim P Tegner Y Wilson DH Randall J Skillback T Pazooki D. Blood biomarkers for brain injury in concussed professional ice hockey players. JAMA Neurol. (2014) 71:684–92. 10.1001/jamaneurol.2014.367 24627036

[52] Rubenstein R Chang B Davies P Wagner AK Robertson CS Wang KK. A novel, ultrasensitive assay for tau: potential for assessing traumatic brain injury in tissues and biofluids. J Neurotrauma. (2015) 32:342–52. 10.1089/neu.2014.3548 25177776 PMC4348038

[53] Rubenstein R Sharma DR Chang B Oumata N Cam M Vaucelle L. Novel mouse tauopathy model for repetitive mild traumatic brain injury: evaluation of long-term effects on cognition and biomarker levels after therapeutic inhibition of tau phosphorylation. Front Neurol. (2019) 10:124. 10.3389/fneur.2019.00124 30915013 PMC6421297

[54] Losurdo M Davidsson J Skold MK. Diffuse axonal injury in the rat brain: axonal injury and oligodendrocyte activity following rotational injury. Brain Sci. (2020) 10:229. 10.3390/brainsci10040229 32290212 PMC7225974

[55] Kulbe JR Hall ED. Chronic traumatic encephalopathy-integration of canonical traumatic brain injury secondary injury mechanisms with tau pathology. Prog Neurobiol. (2017) 158:15–44. 10.1016/j.pneurobio.2017.08.003 28851546 PMC5671903

[56] Marmarou CR Prieto R Taya K Young HF Marmarou A. Marmarou weight drop injury model. In: Chen J Xu ZC Xu XM Zhang JH editors. Animal Models of Acute Neurological Injuries. New York, NY: Springer (2009). p. 393–407. 10.1007/978-1-60327-185-1_34

[57] Bodnar CN Roberts KN Higgins EK Bachstetter AD. A systematic review of closed head injury models of mild traumatic brain injury in mice and rats. J Neurotrauma. (2019) 36:1683–706. 10.1089/neu.2018.6127 30661454 PMC6555186

[58] Griesbach GS Hovda DA Gomez-Pinilla F. Exercise-induced improvement in cognitive performance after traumatic brain injury in rats is dependent on BDNF activation. Brain Res. (2009) 1288:105–15. 10.1016/j.brainres.2009.06.045 19555673 PMC2735616

[59] Griesbach GS Hovda DA Molteni R Wu A Gomez-Pinilla F. Voluntary exercise following traumatic brain injury: brain-derived neurotrophic factor upregulation and recovery of function. Neuroscience. (2004) 125:129–39. 10.1016/j.neuroscience.2004.01.030 15051152

[60] Griesbach GS Tio DL Nair S Hovda DA. Recovery of stress response coincides with responsiveness to voluntary exercise after traumatic brain injury. J Neurotrauma. (2014) 31:674–82. 10.1089/neu.2013.3151 24151829 PMC3961793

[61] Griesbach GS Tio DL Vincelli J McArthur DL Taylor AN. Differential effects of voluntary and forced exercise on stress responses after traumatic brain injury. J Neurotrauma. (2012) 29:1426–33. 10.1089/neu.2011.2229 22233388 PMC3335105

[62] Soltani N Soltani Z Khaksari M Ebrahimi G Hajmohammmadi M Iranpour M. The changes of brain edema and neurological outcome, and the probable mechanisms in diffuse traumatic brain injury induced in rats with the history of exercise. Cell Mol Neurobiol. (2020) 40:555–67. 10.1007/s10571-019-00753-w 31836968 PMC11448905

[63] Mondello S Jeromin A Buki A Bullock R Czeiter E Kovacs N. Glial neuronal ratio: a novel index for differentiating injury type in patients with severe traumatic brain injury. J Neurotrauma. (2012) 29:1096–104. 10.1089/neu.2011.2092 22165978 PMC3325554

[64] Takala RS Posti JP Runtti H Newcombe VF Outtrim J Katila AJ. Glial fibrillary acidic protein and ubiquitin C-terminal hydrolase-L1 as outcome predictors in traumatic brain injury. World Neurosurg. (2016) 87:8–20. 10.1016/j.wneu.2015.10.066 26547005

[65] Zetterberg H Blennow K. Fluid biomarkers for mild traumatic brain injury and related conditions. Nat Rev Neurol. (2016) 12:563–74. 10.1038/nrneurol.2016.127 27632903

[66] Shahim P Politis A van der Merwe A Moore B Chou YY Pham DL. Neurofilament light as a biomarker in traumatic brain injury. Neurology. (2020) 95:e610–e22. 10.1212/WNL.0000000000009983 32641538 PMC7455357

[67] Shahim P Tegner Y Marklund N Blennow K Zetterberg H. Neurofilament light and tau as blood biomarkers for sports-related concussion. Neurology. (2018) 90:e1780–e8. 10.1212/WNL.0000000000005518 29653990 PMC5957307

[68] Papa L Brophy GM Welch RD Lewis LM Braga CF Tan CN. Time course and diagnostic accuracy of glial and neuronal blood biomarkers GFAP and UCH-L1 in a large cohort of trauma patients with and without mild traumatic brain injury. JAMA Neurol. (2016) 73:551–60. 10.1001/jamaneurol.2016.0039 27018834 PMC8805143

[69] Mahan MY Thorpe M Ahmadi A Abdallah T Casey H Sturtevant D. Glial fibrillary acidic protein (GFAP) outperforms S100 calcium-binding protein B (S100B) and ubiquitin C-terminal hydrolase L1 (UCH-L1) as predictor for positive computed tomography of the head in trauma subjects. World Neurosurg. (2019) 128:e434–e44. 10.1016/j.wneu.2019.04.170 31051301

[70] Bernick C Zetterberg H Shan G Banks S Blennow K. Longitudinal performance of plasma neurofilament light and tau in professional fighters: The professional fighters brain health study. J Neurotrauma. (2018) 35:2351–6. 10.1089/neu.2017.5553 29609512

[71] Cheng WH Stukas S Martens KM Namjoshi DR Button EB Wilkinson A. Age at injury and genotype modify acute inflammatory and neurofilament-light responses to mild CHIMERA traumatic brain injury in wild-type and APP/PS1 mice. Exp Neurol. (2018) 301(Pt A):26–38. 10.1016/j.expneurol.2017.12.007 29269117

[72] Cheng WH Martens KM Bashir A Cheung H Stukas S Gibbs E. CHIMERA repetitive mild traumatic brain injury induces chronic behavioural and neuropathological phenotypes in wild-type and APP/PS1 mice. Alzheimers Res Ther. (2019) 11:6. 10.1186/s13195-018-0461-0 30636629 PMC6330571

[73] Pham L Wright DK O'Brien WT Bain J Huang C Sun M. Behavioral, axonal, and proteomic alterations following repeated mild traumatic brain injury: novel insights using a clinically relevant rat model. Neurobiol Dis. (2020) 148:105151. 10.1016/j.nbd.2020.105151 33127468

[74] Gaetani L Blennow K Calabresi P Di Filippo M Parnetti L Zetterberg H. Neurofilament light chain as a biomarker in neurological disorders. J Neurol Neurosurg Psychiatry. (2019) 90:870–81. 10.1136/jnnp-2018-320106 30967444

[75] McKee AC Daneshvar DH. The neuropathology of traumatic brain injury. Handb Clin Neurol. (2015) 127:45–66. 10.1016/B978-0-444-52892-6.00004-0 25702209 PMC4694720

[76] Smith DH Stewart W. 'Concussion' is not a true diagnosis. Nat Rev Neurol. (2020) 16:457–8. 10.1038/s41582-020-0382-y 32647140 PMC7846979

[77] Shahim P Zetterberg H Tegner Y Blennow K. Serum neurofilament light as a biomarker for mild traumatic brain injury in contact sports. Neurology. (2017) 88:1788–94. 10.1212/WNL.0000000000003912 28404801 PMC5419986

[78] Kalm M Bostrom M Sandelius A Eriksson Y Ek CJ Blennow K. Serum concentrations of the axonal injury marker neurofilament light protein are not influenced by blood-brain barrier permeability. Brain Res. (2017) 1668:12–9. 10.1016/j.brainres.2017.05.011 28522263

[79] Farooqui AA. n-3 fatty acid-derived lipid mediators in the brain: new weapons against oxidative stress and inflammation. Curr Med Chem. (2012) 19:532–43. 10.2174/092986712798918851 22204329

[80] Grimm MO Kuchenbecker J Grosgen S Burg VK Hundsdorfer B Rothhaar TL. Docosahexaenoic acid reduces amyloid beta production via multiple pleiotropic mechanisms. J Biol Chem. (2011) 286:14028–39. 10.1074/jbc.M110.182329 21324907 PMC3077603

[81] Eto M Hashimoto T Shimizu T Iwatsubo T. Characterization of the unique in vitro effects of unsaturated fatty acids on the formation of amyloid beta fibrils. PLoS ONE. (2019) 14:e0219465. 10.1371/journal.pone.0219465 31291354 PMC6619765

[82] Belayev L Mukherjee PK Balaszczuk V Calandria JM Obenaus A Khoutorova L. Neuroprotectin D1 upregulates Iduna expression and provides protection in cellular uncompensated oxidative stress and in experimental ischemic stroke. Cell Death Differ. (2017) 24:1091–9. 10.1038/cdd.2017.55 28430183 PMC5442474

[83] Serhan CN Levy BD. Resolvins in inflammation: emergence of the pro-resolving superfamily of mediators. J Clin Invest. (2018) 128:2657–69. 10.1172/JCI97943 29757195 PMC6025982

[84] Lopez-Vicario C Rius B Alcaraz-Quiles J Garcia-Alonso V Lopategi A Titos E. Pro-resolving mediators produced from EPA and DHA: overview of the pathways involved and their mechanisms in metabolic syndrome and related liver diseases. Eur J Pharmacol. (2016) 785:133–43. 10.1016/j.ejphar.2015.03.092 25987424

[85] Bauer I Hughes M Rowsell R Cockerell R Pipingas A Crewther S. Omega-3 supplementation improves cognition and modifies brain activation in young adults. Hum Psychopharmacol. (2014) 29:133–44. 10.1002/hup.2379 24470182

[86] Crenn P Hamchaoui S Bourget-Massari A Hanachi M Melchior JC Azouvi P. Changes in weight after traumatic brain injury in adult patients: a longitudinal study. Clin Nutr. (2014) 33:348–53. 10.1016/j.clnu.2013.06.003 23810396

[87] Howe P Buckley J. Metabolic health benefits of long-chain omega-3 polyunsaturated fatty acids. Mil Med. (2014) 179(11 Suppl.):138–43. 10.7205/MILMED-D-14-00154 25373098

[88] Schober ME Requena DF Abdullah OM Casper TC Beachy J Malleske D. Dietary docosahexaenoic acid improves cognitive function, tissue sparing, and magnetic resonance imaging indices of edema and white matter injury in the immature rat after traumatic brain injury. J Neurotrauma. (2016) 33:390–402. 10.1089/neu.2015.3945 26247583 PMC4761828

[89] Jourdan C Brugel D Hubeaux K Toure H Laurent-Vannier A Chevignard M. Weight gain after childhood traumatic brain injury: a matter of concern. Dev Med Child Neurol. (2012) 54:624–8. 10.1111/j.1469-8749.2012.04291.x 22524689

[90] Mondello S Buki A Barzo P Randall J Provuncher G Hanlon D. CSF and plasma amyloid-beta temporal profiles and relationships with neurological status and mortality after severe traumatic brain injury. Sci Rep. (2014) 4:6446. 10.1038/srep06446 25300247 PMC4192636

[91] van Eijck MM Schoonman GG van der Naalt J de Vries J Roks G. Diffuse axonal injury after traumatic brain injury is a prognostic factor for functional outcome: a systematic review and meta-analysis. Brain Inj. (2018) 32:395–402. 10.1080/02699052.2018.1429018 29381396

[92] Huang XJ Glushakova O Mondello S Van K Hayes RL Lyeth BG. Acute temporal profiles of serum levels of UCH-L1 and GFAP and relationships to neuronal and astroglial pathology following traumatic brain injury in rats. J Neurotrauma. (2015) 32:1179–89. 10.1089/neu.2015.3873 25763798

[93] Szmydynger-Chodobska J Chung I Kozniewska E Tran B Harrington FJ Duncan JA. Increased expression of vasopressin v1a receptors after traumatic brain injury. J Neurotrauma. (2004) 21:1090–102. 10.1089/0897715041651033 15319008

[94] Marmarou CR Liang X Abidi NH Parveen S Taya K Henderson SC. Selective vasopressin-1a receptor antagonist prevents brain edema, reduces astrocytic cell swelling and GFAP, V1aR and AQP4 expression after focal traumatic brain injury. Brain Res. (2014) 1581:89–102. 10.1016/j.brainres.2014.06.005 24933327 PMC4240002

[95] Krieg SM Sonanini S Plesnila N Trabold R. Effect of small molecule vasopressin V1a and V2 receptor antagonists on brain edema formation and secondary brain damage following traumatic brain injury in mice. J Neurotrauma. (2015) 32:221–7. 10.1089/neu.2013.3274 25111427 PMC4321979

[96] Rauen K Pop V Trabold R Badaut J Plesnila N. Vasopressin V1a receptors regulate cerebral aquaporin 1 after traumatic brain injury. J Neurotrauma. (2020) 37:665–74. 10.1089/neu.2019.6653 31547764 PMC7045352

[97] Cui D Jia S Yu J Li D Li T Liu Y. Alleviation of cerebral infarction of rats with middle cerebral artery occlusion by inhibition of aquaporin 4 in the supraoptic nucleus. ASN Neuro. (2020) 12:1759091420960550. 10.1177/1759091420960550 32985231 PMC7545515

[98] Cui D Jia S Li T Li D Wang X Liu X. Alleviation of brain injury by applying TGN-020 in the supraoptic nucleus via inhibiting vasopressin neurons in rats of focal ischemic stroke. Life Sci. (2021) 264:118683. 10.1016/j.lfs.2020.118683 33127515

[99] Hutchinson PJ Kolias AG Tajsic T Adeleye A Aklilu AT Apriawan T. Consensus statement from the international consensus meeting on the role of decompressive craniectomy in the management of traumatic brain injury. Acta Neurochir. (2019) 161:1261–74. 10.1007/s00701-019-03936-y 31134383 PMC6581926

[100] Shriki J Galvagno SM. Sedation for rapid sequence induction and intubation of neurologically injured patients. Emerg Med Clin North Am. (2021) 39:203–16. 10.1016/j.emc.2020.09.012 33218658

[101] Khellaf A Khan DZ Helmy A. Recent advances in traumatic brain injury. J Neurol. (2019) 266:2878–89. 10.1007/s00415-019-09541-4 31563989 PMC6803592

[102] Livingston G Huntley J Sommerlad A Ames D Ballard C Banerjee S. Dementia prevention, intervention, and care: 2020 report of the Lancet Commission. Lancet. (2020) 396:413–46. 10.1016/S0140-6736(20)30367-6 32738937 PMC7392084

