# Supplementary material for: Omega-3 Fatty Acids and Vitamin D Decrease Plasma T-Tau, GFAP, and UCH-L1 in Experimental Traumatic Brain Injury
Source: Front Nutr. 2021 Jun 4;8:685220. doi: 10.3389/fnut.2021.685220 (PMC8211733; doi:10.3389/fnut.2021.685220)
Supplement: Supplementary file 1 [file Data_Sheet_1.PDF]

Supplementary Material

The **Rotarod** is the most efficient and reliable test for vestibular motor activity in the acute post-brain injury phase of up to a week (1). Evoked voluntary motor function before and after exposure to mTBI was assessed in all experimental groups using a 4-station Rotarod treadmill (Model ENV-574R, Med Associates Inc., St Albans, VT). This device has four enclosed lanes of elevated spindles that rotate in parallel with the animal's direction of running. Before head trauma, each animal was trained for 5 days to remain upon the spindle, rotating at a constant velocity (10 rpm) for at least 5 min, to ensure that learning effects did not confound subsequent post-TBI test performance (2). On the day before exposure to the head injury, rats were subjected to a challenge test with the spindle rotating at a steadily accelerating speed from 2.5 to 25 rpm over the 5 min test duration. Post-injury, rats were re-tested on day 2, 7, 14 and 29 to document general motor recovery and the effect of the different diets. For this test, the outcome metric is the latency time that the rat could remain on the Rotarod.

The **Rotating Pole** is a test of coordination and integration of movements (3). While basically the same principal as the Rotarod test (motor function & coordination), this test is more sensitive as it evaluates the ability of the animals to balance and to integrate and coordinate their movements to traverse a horizontal pole, rotating clockwise at 4 rpm and perpendicular to the animal's direction of walking. At one end of the pole (33 mm in diameter, 180 cm in length, and 66 cm off the floor) is a closed box with an entrance hole facing the pole. The floor of the box is covered with bedding material from the home cage of the rat being tested, and thus serves as positive reinforcement for the rat to traverse the pole when placed at the end opposite to the cage. Type E foam bedding was placed on the floor, under the pole, to reduce risk of injury from falls (Foam to Size, Inc.). Rat performance was scored on a numerical rank scale according to the following definitions: Score 1, the animal was unable to balance on the pole and fell off immediately; Score 2, balanced on the pole but has severe difficulty crossing and moved < 30 cm; Score 3, embraced the pole with its paws and managed to move > 30 cm, but did not reach the end; Score 4, traversed the entire pole but embraced it using the paws and/or jumping with the hind legs; Score 5, traversed the entire pole with normal posture but had > 3 foot slips; and Score 6, traversed the entire pole with < 3 foot slips. Whereas latency (mean duration) to simply traverse the pole indicates general ability following training, a high final Rotating pole score indicates that a rat is able to greatly master the task, by reaching a stricter physical performance criterion. To obtain baseline data, two days before exposure to TBI, all animals were trained until they achieved a high performance score of 5 or 6. On Days 2, 7, 15 and 28 after TBI, each animal was tested twice, at 4 rpm. All animals are graded by personnel blinded to the experimental groups of the animals, and subjectivity in the observations was minimized by the involvement of 2 observers in the testing of each animal which came to a common agreement on the assigned scores.

The **Barnes Maze (BM)** is a test of learning and long-term spatial memory, where latency is scored as the "time to locate a hidden box" as previously reported (4). Visual cues in the form of cut out cardboard shapes were placed around the room (on the walls) in plain sight of the rats. A hidden box was provided under one of the 18 holes located around the periphery of the

tabletop (Model ENV-562-R, Med Associates Inc.). Pre-injury, all rats were trained to locate the hidden box over 5 consecutive days. On days 22-26 post-TBI, with the box moved to a new location (approximately 90 degrees away from the first location), latency was again measured over 5 consecutive days. The BM was cleaned of urine and feces with a 30% ethanol solution between testing each rat to prevent the animals from using scent to find the escape box.

References:

1. Hamm RJ. Neurobehavioral assessment of outcome following traumatic brain injury in rats: an evaluation of selected measures. J Neurotrauma. 2001;18(11):1207-16.
2. Arevalo-Martin A, Vela JM, Molina-Holgado E, Borrell J, Guaza C. Therapeutic action of cannabinoids in a murine model of multiple sclerosis. J Neurosci. 2003;23(7):2511-6.
3. Mattiasson GJ, Philips MF, Tomasevic G, Johansson BB, Wieloch T, McIntosh TK. The rotating pole test: evaluation of its effectiveness in assessing functional motor deficits following experimental head injury in the rat. J Neurosci Methods. 2000;95(1):75-82.
4. Rosenfeld CS, Ferguson SA. Barnes maze testing strategies with small and large rodent models. J Vis Exp. 2014;84:e51194.
